# Supplementary material for: In vitro cytotoxicity (irritant potency) of toothpaste ingredients
Source: PLoS One. 2025 Jan 30;20(1):e0318565. doi: 10.1371/journal.pone.0318565 (PMC11781688; doi:10.1371/journal.pone.0318565)
Supplement: Attachment — Submitted filename: Response to Reviewers.docx [file pone.0318565.s002.docx]

Dear editor and reviewers,

First of all, we would like to thank you for the extremely careful review. During my long academic career, I have never received such a carefully executed review! It has also motivated us to look at the text even more carefully and make improvements.

Thanks to your feedback, the article has improved.

In the text below, our answers to each comment are indicated in red.

We look forward to your decision.

Kind regards, also on behalf of the co-authors

Albert J. Feilzer

PONE-D-24-31912

In vitro cytotoxicity (irritant potency) of toothpaste ingredients

PLOS ONE

Dear Dr.  Feilzer,

Thank you for submitting your manuscript to PLOS ONE. After careful consideration, we feel that it has merit but does not fully meet PLOS ONE’s publication criteria as it currently stands. Therefore, we invite you to submit a revised version of the manuscript that addresses the points raised during the review process.

Please submit your revised manuscript by Oct 26 2024 11:59PM. If you will need more time than this to complete your revisions, please reply to this message or contact the journal office at [plosone@plos.org](mailto:plosone@plos.org). When you're ready to submit your revision, log on to <https://www.editorialmanager.com/pone/> and select the 'Submissions Needing Revision' folder to locate your manuscript file.

Please include the following items when submitting your revised manuscript:

- A rebuttal letter that responds to each point raised by the academic editor and reviewer(s). You should upload this letter as a separate file labeled 'Response to Reviewers'.
- A marked-up copy of your manuscript that highlights changes made to the original version. You should upload this as a separate file labeled 'Revised Manuscript with Track Changes'.
- An unmarked version of your revised paper without tracked changes. You should upload this as a separate file labeled 'Manuscript'.

If you would like to make changes to your financial disclosure, please include your updated statement in your cover letter. Guidelines for resubmitting your figure files are available below the reviewer comments at the end of this letter.

If applicable, we recommend that you deposit your laboratory protocols in protocols.io to enhance the reproducibility of your results. Protocols.io assigns your protocol its own identifier (DOI) so that it can be cited independently in the future. For instructions see: [https://journals.plos.org/plosone/s/submission-guidelines#loc-laboratory-protocols](https://c05y1x9s.r.us-east-2.awstrack.me/L0/https:%2F%2Fjournals.plos.org%2Fplosone%2Fs%2Fsubmission-guidelines%23loc-laboratory-protocols/1/010f0191e3fa8dda-b36a2262-5fa4-40b6-b241-55c8589150e0-000000/UeCF3zAooY_Cmo9yNpQmkph8Tt0=176). Additionally, PLOS ONE offers an option for publishing peer-reviewed Lab Protocol articles, which describe protocols hosted on protocols.io. Read more information on sharing protocols at [https://plos.org/protocols?utm_medium=editorial-email&utm_source=authorletters&utm_campaign=protocols](https://c05y1x9s.r.us-east-2.awstrack.me/L0/https:%2F%2Fplos.org%2Fprotocols%3Futm_medium=editorial-email%26utm_source=authorletters%26utm_campaign=protocols/1/010f0191e3fa8dda-b36a2262-5fa4-40b6-b241-55c8589150e0-000000/kuyxVr9vACNrl02XUX0qrnvW6Oo=176).

We look forward to receiving your revised manuscript.

Kind regards,

Tanay Chaubal
Academic Editor
PLOS ONE

Journal Requirements:

When submitting your revision, we need you to address these additional requirements. 

[1. Please ensure that your manuscript meets PLOS ONE's style requirements, including those for file naming. The PLOS ONE style templates can be found at https://journals.plos.org/plosone/s/file?id=wjVg/PLOSOne_formatting_sample_main_body.pdf and](1.%20Please%20ensure%20that%20your%20manuscript%20meets%20PLOS%20ONE's%20style%20requirements,%20including%20those%20for%20file%20naming.%20The%20PLOS%20ONE%20style%20templates%20can%20be%20found%20at%20https://journals.plos.org/plosone/s/file?id=wjVg/PLOSOne_formatting_sample_main_body.pdf%20and%20) [https://journals.plos.org/plosone/s/file?id=ba62/PLOSOne_formatting_sample_title_authors_affiliations.pdf](https://c05y1x9s.r.us-east-2.awstrack.me/L0/https:%2F%2Fjournals.plos.org%2Fplosone%2Fs%2Ffile%3Fid=ba62%2FPLOSOne_formatting_sample_title_authors_affiliations.pdf/1/010f0191e3fa8dda-b36a2262-5fa4-40b6-b241-55c8589150e0-000000/PZyY_E58qSUX7iAHvlCKdiWGdTw=176)

2.  We note that your Data Availability Statement is currently as follows: “All relevant data are within the manuscript and its Supporting Information files.”

The data are added as supplementary file: MTT data-file.xlsx

Please confirm at this time whether or not your submission contains all raw data required to replicate the results of your study. Authors must share the “minimal data set” for their submission. PLOS defines the minimal data set to consist of the data required to replicate all study findings reported in the article, as well as related metadata and methods (<https://journals.plos.org/plosone/s/data-availability#loc-minimal-data-set-definition>). For example, authors should submit the following data: - The values behind the means, standard deviations and other measures reported; - The values used to build graphs; - The points extracted from images for analysis. Authors do not need to submit their entire data set if only a portion of the data was used in the reported study. If your submission does not contain these data, please either upload them as Supporting Information files or deposit them to a stable, public repository and provide us with the relevant URLs, DOIs, or accession numbers. For a list of recommended repositories, please see <https://journals.plos.org/plosone/s/recommended-repositories>. If there are ethical or legal restrictions on sharing a de-identified data set, please explain them in detail (e.g., data contain potentially sensitive information, data are owned by a third-party organization, etc.) and who has imposed them (e.g., an ethics committee). Please also provide contact information for a data access committee, ethics committee, or other institutional body to which data requests may be sent. If data are owned by a third party, please indicate how others may request data access.

3. [Please include captions for your Supporting Information files at the end of your manuscript, and update any in-text citations to match accordingly. Please see our Supporting Information guidelines for more information: http://journals.plos.org/plosone/s/supporting-information.](https://vunl-my.sharepoint.com/personal/a_feilzer_acta_nl/Documents/Please%20include%20captions%20for%20your%20Supporting%20Information%20files%20at%20the%20end%20of%20your%20manuscript,%20and%20update%20any%20in-text%20citations%20to%20match%20accordingly.%20Please%20see%20our%20Supporting%20Information%20guidelines%20for%20more%20information:%20http:/journals.plos.org/plosone/s/supporting-information.)

We include the captions of the supporting Information files at the end of our manuscript, and update any in-text citations to match accordingly.

4. Please review your reference list to ensure that it is complete and correct. If you have cited papers that have been retracted, please include the rationale for doing so in the manuscript text, or remove these references and replace them with relevant current references. Any changes to the reference list should be mentioned in the rebuttal letter that accompanies your revised manuscript. If you need to cite a retracted article, indicate the article’s retracted status in the References list and also include a citation and full reference for the retraction notice.

Additional Editor Comments:
 **I thank you for submitting an interesting manuscript. However, there are comments made by reviewers which potentially could make your manuscript more robust.**

[Note: HTML markup is below. Please do not edit.]

Dear Prof. Chaubal,

Please find below our answers to the queries highlighted in red:

Reviewers' comments:

Reviewer's Responses to Questions

**Comments to the Author**

1. Is the manuscript technically sound, and do the data support the conclusions?

The manuscript must describe a technically sound piece of scientific research with data that supports the conclusions. Experiments must have been conducted rigorously, with appropriate controls, replication, and sample sizes. The conclusions must be drawn appropriately based on the data presented.

Reviewer #1: Yes

Reviewer #2: Yes

2. Has the statistical analysis been performed appropriately and rigorously?

Reviewer #1: N/A

Reviewer #2: N/A

3. Have the authors made all data underlying the findings in their manuscript fully available?

The [PLOS Data policy](http://c05y1x9s.r.us-east-2.awstrack.me/L0/http:%2F%2Fwww.plosone.org%2Fstatic%2Fpolicies.action%23sharing/1/010f0191e3fa8dda-b36a2262-5fa4-40b6-b241-55c8589150e0-000000/iya5eEiaQSM0Pko1Dz8IysdHdn4=176) requires authors to make all data underlying the findings described in their manuscript fully available without restriction, with rare exception (please refer to the Data Availability Statement in the manuscript PDF file). The data should be provided as part of the manuscript or its supporting information, or deposited to a public repository. For example, in addition to summary statistics, the data points behind means, medians and variance measures should be available. If there are restrictions on publicly sharing data—e.g. participant privacy or use of data from a third party—those must be specified.

Reviewer #1: No

Original raw data are uploaded as supplementary file

Reviewer #2: Yes

4. Is the manuscript presented in an intelligible fashion and written in standard English?

PLOS ONE does not copyedit accepted manuscripts, so the language in submitted articles must be clear, correct, and unambiguous. Any typographical or grammatical errors should be corrected at revision, so please note any specific errors here.

Reviewer #1: Yes

Reviewer #2: Yes

5. Review Comments to the Author

Please use the space provided to explain your answers to the questions above. You may also include additional comments for the author, including concerns about dual publication, research ethics, or publication ethics. (Please upload your review as an attachment if it exceeds 20,000 characters)

Reviewer #1: The study highlights the potential for certain toothpaste ingredients to harm the cells. The strength of the paper is the use of a bilayered RHG model, which enhances the relevance of the findings to human oral health. However, the study's limitation lies in the inability to directly compare in vitro concentrations with those in commercial toothpaste, which could affect the interpretation of the results. These are stated as limitation in the manuscript.

Authors are requested to make certain clarifications in the paper.

1. Were the experiments conducted in triplicates/duplicates? Were the data from MTT values normalized? before comparing? If not, it is advised to show the data (MTT observations) properly in a table form.

The experiments are conducted in duplicate.

The data from the MTT are per culture and test material compared to that of unexposed RHG of the same culture as well as to the values of exposure to the test vehicle only. The data are ‘normalized’ to the results of the exposure to the test vehicle per culture.

Clarification is added to the text:

“All experiments were conducted in two independent experiments, each using a different batch of RHG which had been grown separately at different times.”

“The data from the MTT assay are per RHG exposed to a test material compared to that of unexposed RHG of the same RHG batch as well as to the values obtained from exposure to the test vehicle only. The data are shown normalized to the results of the exposure to the test vehicle per culture.”

2. What statistical analyses was performed? please state and show statistical values legibly.

Data in duplicate are reported as average and standard deviation. The calculation of the EC10 and EC 50 values was done by 4-point logistic regression.

The text is changed: “Values were obtained by linear regression 4 point logistic analysis based on changes in metabolic activity (MTT). The web-based EC50 Calculator of AAT Bioquest [28] was used.”

3. The changes in the histology is not mentioned in detail anywhere in the manuscript. It is required to state and show observed changes clearly. What are the changes observed.

The observed changes are added to the figure legend (Fig. 3)

4. Photographs depicting the RHG model and preparation will benefit readers. it is recommended to add them.

A figure (Fig. 1) depicting the RHG model in a MTT is added to the manuscript.

5. The discussion part can be improved in terms of comparing the observations to existing evidences in the literature rather than just reporting the observations.

The plan involved initially assessing the toxicity of all potentially harmful ingredients in toothpaste using the MTT assay based on the RHG model. After this study, we will begin testing toothpastes containing these ingredients. The current results reflect a single exposure to the test materials. For instance, when testing a toothpaste, these substances may either attenuate or potentiate each other’s effects. Additionally, we emphasized that our MTT assay was conducted in an RHG model rather than with single cell types. Therefore, we decided not to compare our observations with the existing scientific literature at this stage. Furthermore, we have already demonstrated that the RHG model produces similar tissue responses when exposed to materials, as shown in Buskermolen’s study.

Reviewer #2: The aim and purpose of the work is very well exposed. This study reminds so interesting and have got and excellent clinical transpolation. Please describe in more detail the statistical analysis used.

After the thorough review of the PLOS reviewers and during answering their questions, we realized that the linear regression used on the MTT data was outdated. Consequently, we reanalyzed the data using a 4-point logistic analysis. We replaced the bar graphs ( Fig. 2) with logistic regression plots, which offer better insights into the slope differences between the test materials.

We have clarified in the text that the 4-point logistic analysis was performed using ATT’s web-based calculator. All necessary data to reproduce the analysis have been included in a supplementary file.

6. PLOS authors have the option to publish the peer review history of their article ([what does this mean?](https://c05y1x9s.r.us-east-2.awstrack.me/L0/https:%2F%2Fjournals.plos.org%2Fplosone%2Fs%2Feditorial-and-peer-review-process%23loc-peer-review-history/1/010f0191e3fa8dda-b36a2262-5fa4-40b6-b241-55c8589150e0-000000/uSX93XnjpVyQ1tsJG53yX7IKKws=176)). If published, this will include your full peer review and any attached files.


If you choose “no”, your identity will remain anonymous but your review may still be made public.


**Do you want your identity to be public for this peer review?** For information about this choice, including consent withdrawal, please see our [Privacy Policy](https://c05y1x9s.r.us-east-2.awstrack.me/L0/https:%2F%2Fwww.plos.org%2Fprivacy-policy/1/010f0191e3fa8dda-b36a2262-5fa4-40b6-b241-55c8589150e0-000000/JpTqEiBOoaxUFS52gBT-A8cuUes=176).

Reviewer #1: **Yes:** Spoorthi Ravi Banavar

Reviewer #2: **Yes:** Evelin Bachmeier

[NOTE: If reviewer comments were submitted as an attachment file, they will be attached to this email and accessible via the submission site. Please log into your account, locate the manuscript record, and check for the action link "View Attachments". If this link does not appear, there are no attachment files.]

While revising your submission, please upload your figure files to the Preflight Analysis and Conversion Engine (PACE) digital diagnostic tool, [https://pacev2.apexcovantage.com/](https://c05y1x9s.r.us-east-2.awstrack.me/L0/https:%2F%2Fpacev2.apexcovantage.com%2F/1/010f0191e3fa8dda-b36a2262-5fa4-40b6-b241-55c8589150e0-000000/jexTL0cD6HDj5JPafdDnzdHerSw=176). PACE helps ensure that figures meet PLOS requirements. To use PACE, you must first register as a user. Registration is free. Then, login and navigate to the UPLOAD tab, where you will find detailed instructions on how to use the tool. If you encounter any issues or have any questions when using PACE, please email PLOS at [figures@plos.org](mailto:figures@plos.org). Please note that Supporting Information files do not need this step.

*In compliance with data protection regulations, you may request that we remove your personal registration details at any time.* [*(Remove my information/details)*](https://c05y1x9s.r.us-east-2.awstrack.me/L0/https:%2F%2Fwww.editorialmanager.com%2Fpone%2Flogin.asp%3Fa=r/1/010f0191e3fa8dda-b36a2262-5fa4-40b6-b241-55c8589150e0-000000/mFgtL9BCVuwVZEqM5n0b0hhq_5k=176)*. Please contact the publication office if you have any questions.*

Kind regards,

Albert J. Feilzer
