# Supplementary material for: In vitro cytotoxicity (irritant potency) of toothpaste ingredients
Source: PLoS One. 2025 Jan 30;20(1):e0318565. doi: 10.1371/journal.pone.0318565 (PMC11781688; doi:10.1371/journal.pone.0318565)
Supplement: Attachment — Submitted filename: Response to the reviewers 9-1-25.docx [file pone.0318565.s003.docx]

PONE-D-24-31912R1

In vitro cytotoxicity (irritant potency) of toothpaste ingredients

PLOS ONE

Dear Dr. Feilzer,

Thank you for submitting your manuscript to PLOS ONE. After careful consideration, we feel that it has merit but does not fully meet PLOS ONE’s publication criteria as it currently stands. Therefore, we invite you to submit a revised version of the manuscript that addresses the points raised during the review process.

==============================
**ACADEMIC EDITOR: I thank you for responding to the reviewers' comments. However, there still minor revisions which are required as per reviewers' comments.**
==============================

Please submit your revised manuscript by Feb 10 2025 11:59PM. If you will need more time than this to complete your revisions, please reply to this message or contact the journal office at [plosone@plos.org](mailto:plosone@plos.org). When you're ready to submit your revision, log on to <https://www.editorialmanager.com/pone/> and select the 'Submissions Needing Revision' folder to locate your manuscript file.

Please include the following items when submitting your revised manuscript:

- A rebuttal letter that responds to each point raised by the academic editor and reviewer(s). You should upload this letter as a separate file labeled 'Response to Reviewers'.
- A marked-up copy of your manuscript that highlights changes made to the original version. You should upload this as a separate file labeled 'Revised Manuscript with Track Changes'.
- An unmarked version of your revised paper without tracked changes. You should upload this as a separate file labeled 'Manuscript'.

If you would like to make changes to your financial disclosure, please include your updated statement in your cover letter. Guidelines for resubmitting your figure files are available below the reviewer comments at the end of this letter.

If applicable, we recommend that you deposit your laboratory protocols in protocols.io to enhance the reproducibility of your results. Protocols.io assigns your protocol its own identifier (DOI) so that it can be cited independently in the future. For instructions see: [https://journals.plos.org/plosone/s/submission-guidelines#loc-laboratory-protocols](https://track.editorialmanager.com/CL0/https:%2F%2Fjournals.plos.org%2Fplosone%2Fs%2Fsubmission-guidelines%23loc-laboratory-protocols/1/010f019407513574-426b0b61-6a0e-4e01-947e-4ae69b7c1c6c-000000/yg1g40C218heqEX1Hv1vls6nA8DKwMC7CFGMSeWBAqE=190). Additionally, PLOS ONE offers an option for publishing peer-reviewed Lab Protocol articles, which describe protocols hosted on protocols.io. Read more information on sharing protocols at [https://plos.org/protocols?utm_medium=editorial-email&utm_source=authorletters&utm_campaign=protocols](https://track.editorialmanager.com/CL0/https:%2F%2Fplos.org%2Fprotocols%3Futm_medium=editorial-email%26utm_source=authorletters%26utm_campaign=protocols/1/010f019407513574-426b0b61-6a0e-4e01-947e-4ae69b7c1c6c-000000/kL7h-2_NKWR6yrEdL3OOun8erWXg7MwvNEm_TknOIWE=190).

We look forward to receiving your revised manuscript.

Kind regards,

Tanay Chaubal
Academic Editor
PLOS ONE

Journal Requirements:

Please review your reference list to ensure that it is complete and correct. If you have cited papers that have been retracted, please include the rationale for doing so in the manuscript text, or remove these references and replace them with relevant current references. Any changes to the reference list should be mentioned in the rebuttal letter that accompanies your revised manuscript. If you need to cite a retracted article, indicate the article’s retracted status in the References list and also include a citation and full reference for the retraction notice.


[Note: HTML markup is below. Please do not edit.]

Reviewers' comments:

Reviewer's Responses to Questions

**Comments to the Author**

1. If the authors have adequately addressed your comments raised in a previous round of review and you feel that this manuscript is now acceptable for publication, you may indicate that here to bypass the “Comments to the Author” section, enter your conflict of interest statement in the “Confidential to Editor” section, and submit your "Accept" recommendation.

Reviewer #1: All comments have been addressed

Reviewer #2: All comments have been addressed

2. Is the manuscript technically sound, and do the data support the conclusions?

The manuscript must describe a technically sound piece of scientific research with data that supports the conclusions. Experiments must have been conducted rigorously, with appropriate controls, replication, and sample sizes. The conclusions must be drawn appropriately based on the data presented.

Reviewer #1: Yes

Reviewer #2: Yes

3. Has the statistical analysis been performed appropriately and rigorously?

Reviewer #1: Yes

Reviewer #2: Yes

4. Have the authors made all data underlying the findings in their manuscript fully available?

The [PLOS Data policy](http://track.editorialmanager.com/CL0/http:%2F%2Fwww.plosone.org%2Fstatic%2Fpolicies.action%23sharing/1/010f019407513574-426b0b61-6a0e-4e01-947e-4ae69b7c1c6c-000000/KaLOSrCzmwpDKXSwNr4IeoGPWRjEIVfDclYO10p5WIs=190) requires authors to make all data underlying the findings described in their manuscript fully available without restriction, with rare exception (please refer to the Data Availability Statement in the manuscript PDF file). The data should be provided as part of the manuscript or its supporting information, or deposited to a public repository. For example, in addition to summary statistics, the data points behind means, medians and variance measures should be available. If there are restrictions on publicly sharing data—e.g. participant privacy or use of data from a third party—those must be specified.

Reviewer #1: Yes

Reviewer #2: Yes

5. Is the manuscript presented in an intelligible fashion and written in standard English?

PLOS ONE does not copyedit accepted manuscripts, so the language in submitted articles must be clear, correct, and unambiguous. Any typographical or grammatical errors should be corrected at revision, so please note any specific errors here.

Reviewer #1: Yes

Reviewer #2: Yes

6. Review Comments to the Author

Please use the space provided to explain your answers to the questions above. You may also include additional comments for the author, including concerns about dual publication, research ethics, or publication ethics. (Please upload your review as an attachment if it exceeds 20,000 characters)

Reviewer #1: Thank you for addressing our comments. The manuscript describes the methods and results as required. However, it is recommended to always perform experiments in triplicates and not duplicates. Additionally, I would like to point that the standard deviations noted in the experiments are very wide and this is a point of concern especially when done in duplicates. If this cannot be addressed now, please mention in the limitation. Additionally, it is recommended to depict the pictures of the RHG model before and during histology. this will benefit readers.

***We add the following text to the discussion section:***

*The MTT values exhibited for certain chemicals a significant variability at specific concentrations, particularly in the steep sections of the graphs, leading to high standard deviations. However, since the X-axis is on a logarithmic scale and is used to determine EC10 and EC50 values, this variability does not impact the final potency scoring. This study followed a previously established standard operating procedure for the Epidermal Equivalent potency assay.[29] This procedure outlines how to perform dose-finding for any unknown coded chemical (with unknown molecular mass) to obtain an EC50 value by conducting two independent experiments. We previously demonstrated that this methodology is transferable to various commercially available and in-house reconstructed skin models, even in international ring studies. [6] Now, we show that it is not only suitable for assessing skin irritant potency in reconstructed human skin models but can also be applied to reconstructed human gingival models. Additionally we changed the figure 3 in accordance with the suggestion of the reviewer.*

Reviewer #2: All request have been answered.
Recommendatons have been attended. The article is very interesting. Ethical aspects have been taken into account.

7. PLOS authors have the option to publish the peer review history of their article ([what does this mean?](https://track.editorialmanager.com/CL0/https:%2F%2Fjournals.plos.org%2Fplosone%2Fs%2Feditorial-and-peer-review-process%23loc-peer-review-history/1/010f019407513574-426b0b61-6a0e-4e01-947e-4ae69b7c1c6c-000000/pbByArEbO-899ymPXlQgUEJ8LBRXsM3lEAIpoy6xhUE=190)). If published, this will include your full peer review and any attached files.


If you choose “no”, your identity will remain anonymous but your review may still be made public.


**Do you want your identity to be public for this peer review?** For information about this choice, including consent withdrawal, please see our [Privacy Policy](https://track.editorialmanager.com/CL0/https:%2F%2Fwww.plos.org%2Fprivacy-policy/1/010f019407513574-426b0b61-6a0e-4e01-947e-4ae69b7c1c6c-000000/Xuw2IizNHfQv-O-VOlaV-M5LQGzMlBjdU-PRzEJeUFI=190).

Reviewer #1: **Yes:** Spoorthi Ravi Banavar

Reviewer #2: **Yes:** Evelin Bachmeier

[NOTE: If reviewer comments were submitted as an attachment file, they will be attached to this email and accessible via the submission site. Please log into your account, locate the manuscript record, and check for the action link "View Attachments". If this link does not appear, there are no attachment files.]

While revising your submission, please upload your figure files to the Preflight Analysis and Conversion Engine (PACE) digital diagnostic tool, [https://pacev2.apexcovantage.com/](https://track.editorialmanager.com/CL0/https:%2F%2Fpacev2.apexcovantage.com%2F/1/010f019407513574-426b0b61-6a0e-4e01-947e-4ae69b7c1c6c-000000/0_qMis56OpEuv2ajgmGdarzjFe0YU-FyDwsEYbFZntI=190). PACE helps ensure that figures meet PLOS requirements. To use PACE, you must first register as a user. Registration is free. Then, login and navigate to the UPLOAD tab, where you will find detailed instructions on how to use the tool. If you encounter any issues or have any questions when using PACE, please email PLOS at [figures@plos.org](mailto:figures@plos.org). Please note that Supporting Information files do not need this step.

*In compliance with data protection regulations, you may request that we remove your personal registration details at any time.* [*(Remove my information/details)*](https://track.editorialmanager.com/CL0/https:%2F%2Fwww.editorialmanager.com%2Fpone%2Flogin.asp%3Fa=r/1/010f019407513574-426b0b61-6a0e-4e01-947e-4ae69b7c1c6c-000000/2cHbnDFRM0SsiDme4GP3Zo8bRNfUWji3fqDZn5JvhSU=190)*. Please contact the publication office if you have any questions.*
